# Multifaceted roles of ninjurin1 in immunity, cell death, and disease

**DOI:** 10.3389/fimmu.2025.1519519

**Published:** 2025-01-31

**Authors:** Lili Zhu, Yunfei Xu

**Affiliations:** ^1^ Department of Pathology, The Affiliated Cancer Hospital of Xiangya School of Medicine, Central South University/Hunan Cancer Hospital, Changsha, China; ^2^ Department of Pathophysiology, School of Basic Medical Sciences, Central South University, Changsha, Hunan, China; ^3^ Postdoctoral Research Station of Biology, School of Basic Medical Science, Central South University, Changsha, Hunan, China

**Keywords:** cancer, inflammation, lytic cell death, neurological diseases, ninjurin1, plasma membrane rupture

## Abstract

Ninjurin1 (NINJ1) is initially identified as a nerve injury-induced adhesion molecule that facilitates axon growth. It is initially characterized to promote nerve regeneration and mediate the transendothelial transport of monocytes/macrophages associated with neuroinflammation. Recent evidence indicates that NINJ1 mediates plasma membrane rupture (PMR) in lytic cell death. The absence or inhibition of NINJ1 can delay PMR, thereby mitigating the spread of inflammation resulting from cell lysis and preventing the progression of various cell death-related pathologies, suggesting a conserved regulatory mechanism across these processes. Further research elucidated the structural basis and mechanism of NINJ1-mediated PMR. Although the role of NINJ1 in PMR is established, the identity of its activating factors and its implications in diseases remain to be fully explored. This review synthesizes current knowledge regarding the structural basis and mechanism of NINJ1-mediated PMR and discusses its significance and therapeutic targeting potential in inflammatory diseases, neurological disorders, cancer, and vascular injuries.

Nerve injury-induced protein 1 (NINJ1) was initially identified as a novel adhesion molecule in the central nervous system (CNS), facilitating cell adhesion through homophilic binding (1). Following axotomy, NINJ1 is upregulated in neurons and Schwann cells at the distal nerve segment, promoting neurite outgrowth. Subsequently, further studies demonstrated that NINJ1 also participates in neuroinflammation (2, 3), angiogenesis (4), and repetitive and anxiety behaviors of neuropsychiatric disorders (5) in CNS. Meanwhile, the crucial role of NINJ1 in cardiovascular diseases, diabetes, inflammation, and cancer has also been elucidated. Despite these findings, NINJ1 did not attract significant attention until 2021, when Kayagaki et al. revealed its role in mediating plasma membrane rupture (PMR) during lytic cell death, thus thrusting NINJ1 into the realm of cell death (6). Subsequent investigations have delineated the structural basis and mechanisms underlying NINJ1-mediated PMR (7). Consequently, the novel role of NINJ1 in cell death has propelled it into a pivotal position, yet its precise contributions to cell death and disease remain partially unclear. Hence, this review aims to shed light on the characteristics of NINJ1 and its connections to cell death and disease, laying a foundation for future comprehensive studies on NINJ1.

## 1 The expression and distribution of NINJ1

NINJ1 was originally discovered in the CNS and is predominantly expressed in neurons and Schwann cells surrounding the distal nerve segment (1). Additionally, Ahn et al. and Ifergan et al. observed that NINJ1 is mainly expressed in the meninges, the choroid plexus, and the parenchymal perivascular region of normal rat brains, while weakly expressed in the cerebral cortex and hippocampal regions, and is absent in lymphoid and astrocytes (8, 9). Subsequently, Lee et al. expanded on the expression and distribution of NINJ1 in CNS (10). They examined the expression profile and cell distribution of NINJ1 in the brain of rats with transient focal cerebral ischemia and found that the expression of NINJ1 was significantly induced in the cortical penumbral area and the cerebral infarction nucleus area on the 1st day after middle cerebral artery occlusion (MCAO). The expression of NINJ1 in the former was high and then decreased after 8 days, while the expression in the latter was maintained for 10 days. On day 1 after MCAO, NINJ1 was detected mainly in the infarct center and penumbral area neutrophils and endothelial cells, but on day 4 after MCAO, reactive macrophages were the primary expression cells of NINJ1. At 12 days after MCAO, expression induction of reactive macrophages was maintained in the infarct center but not in the penumbral area. NINJ1 exhibited dynamic expression patterns in different immune cells at different times after MCAO, suggesting that the function of NINJ1 may also be similarly dynamic. In 2019, Shin et al. investigated the localization of NINJ1 in various tissues using immunohistochemical analysis and Western Blot analysis. They detected the weakest expression of NINJ1 in the brain, which was only detected in a few glial cells, while it was abundant in skin and ileum, and moderately expressed in the sciatic nerve, spleen, lung, stomach, colon, liver, pancreas, kidney, testis and other tissues (11). Besides, according to the data of NIH-NCBI-Gene database (Gene ID: 4814), INJ1 is most abundantly expressed in the kidney, followed by the adrenal gland, thyroid gland, lung, heart, spleen, and liver. Other commonly expressed tissues include the brain, placenta, prostate, salivary gland, small intestine, stomach, thymus, uterus, trachea, and skeletal muscle. It is worth noting that although low expression of NINJ1 still exerts an important role in the CNS, its role in other organs with medium or high expression remains unclear. Hence, more experiments are necessary to uncover the specific molecular mechanisms involving NINJ1 in each organ system. Additionally, NINJ1 was also expressed in epithelial cells, blood cells (1), and myeloid cells (8). According to the data of the scRNASeqDB (https://bioinfo.uth.edu), NINJ1 is most expressed in macrophages, followed by HepG2 cell line, DG-75, liver cancer cell, pancreatic islet duct and so on. Immunofluorescence imaging has demonstrated that NINJ1 is specifically localized within the Golgi apparatus (7). In another study, it was observed that NINJ1 expression encircles both the cell membrane and the nucleus, resembling either the endoplasmic reticulum or the Golgi apparatus in morphology (12). However, it remains to be clarified whether the presence of NINJ1 in these membrane-bound organelles is its original site of expression or a consequence of its activation and subsequent secretion pathway.

## 2 The structural basis and modification of NINJ1

NINJ1 is a 16-kDa plasma membrane protein that is evolutionarily conserved not only between rodents and humans (with an identity of 89.5% and a homology of 95.4%), but also in vertebrates and can even be found in all higher eukaryotes (7, 13). The human NINJ1 gene harbors an open reading frame that encodes a polypeptide consisting of 152 amino acids, comprising two transmembrane regions, one intracellular region, and two extracellular regions located at the N-terminus and C-terminus, respectively (13). NINJ1 features two hydrophobic transmembrane domains, positioned at amino acids 72 to 100 and 118 to 139, as documented by Butcher and Picker (14). Moreover, the pivotal homophilic and heterophilic adhesion domain of NINJ1 is situated within a 12-residue segment spanning from Pro26 to Asn37 in the Extracellular N-Terminal (ENT) domain. This region is characterized by the presence of tryptophan and a contiguous cluster of arginine residues, as identified by Araki et al. (15). The ENT domain, equipped with this crucial adhesion domain, plays a pivotal role in cell-cell interactions and is hypothesized to function as an intrinsic negative regulator of the immune response, potentially via homophilic binding mechanisms (16).

As a cell surface protein belonging to the isotropic adhesion molecule family, NINJ1 exhibits cross-interaction between immune cells and endothelial cells. Importantly, this interaction is intricately associated with the modification and structural attributes of NINJ1 (13). Specifically, NINJ1 assembles into a homocomplex comprising 2 to 6 NINJ1 monomers through a cis-facilitated interaction between its intracellular domain and the N-glycosylation of Asn60. This intricate assembly process is further enabled by intracellular fragments spanning Leu101 to Ala110, which play a pivotal role in the aggregation of NINJ1 homologs. Notably, N-glycosylation is indispensable for the cis-interaction of NINJ1, and any disruption of this glycosylation site, such as through Asn substitution, impedes the formation of NINJ1 homocomplexes. The conserved sequences within NINJ1 include both an N-glycosylation motif and an intracellular region crucial for cis-interaction, and both are essential for the successful assembly of NINJ1 homologs. These findings highlight the critical role of N-glycosylation in the structural integrity and assembly of NINJ1 into homologous protein complexes.

The novel role of NINJ1 in mediating cell death has prompted researchers to delve into the structural basis of NINJ1-mediated PMR during this process, thus shedding new light on the intricate mechanisms of cell death (7). The initial 38 amino acids of the hNINJ1 protein retain a disordered conformation. However, the subsequent 103 amino acids, spanning positions 39 to 141, are distinctly depicted in the structural maps, enabling their unambiguous modeling into four distinct α-helices, designated α1 through α4. In viable, quiescent cells, NINJ1 exists as a monomer anchored to the cell membrane, with α1 and α2 helices protruding towards the extracellular milieu, while the α3 and α4 helices are integral components embedded within the membrane. Upon activation of the inflammasome, triggering a process termed pyroptosis, there is a sequential aggregation of NINJ1 (7). The emergence of NINJ1 dimers and trimers within 10 minutes after inflammasome activation heralds a cascade of aggregation leading to the formation of larger, more complex polymers at subsequent time intervals. NINJ1 congregates on the cell membrane and subsequently polymerizes to form filaments. These aggregates take on various configurations, such as large, branching clusters and elongated filaments of diverse shapes, often extending to the micron scale. During the process of lytic cell death, the extracellular α-helices of NINJ1 insert into the plasma membrane, facilitating the polymerization of NINJ1 monomers into amphipathic filaments. These filaments can encircle the membrane edges in various configurations, ultimately resulting in the rupture of the plasma membrane. Meanwhile, the cryogenic electron microscopy (cryo-EM) structure of NINJ1 reveals another unique way of mediating PMR, namely that NINJ1 dissolves liposomes and forms ring-like structures in activated macrophages to cut and shed membrane disks to mediate PMR (17). Later, Sahoo et al. used cryo-EM to resolve the structures of NINJ1 and its close paralog NINJ2, which is unable to mediate PMR, in order to determine and compare the structures of NINJ1 and NINJ2 (18). This provided mechanistic insights into NINJ1-mediated PMR. They showed that both NINJ1 and NINJ2 assemble into linear filaments that are hydrophobic on one side and hydrophilic on the other. This structural characteristic, along with other evidence, suggests a PMR mechanism where NINJ1 filaments wrap around and solubilize membrane fragments and, less commonly, form pores in the plasma membrane. In contrast to the straight NINJ1 filament, the NINJ2 filament curves towards the intracellular space. This curvature prevents its circularization or even assembly on a relatively flat membrane, thus precluding it from mediating PMR. Mutagenesis studies further demonstrated that the curvature of the NINJ2 filament is induced by its strong association with lipids, particularly a cholesterol molecule, at the cytoplasmic leaflet of the lipid bilayer (18). These researches have enhanced our understanding of the molecular processes by which the role of NINJ1 governs programmed cell death.

## 3 NINJ1 mediates plasma membrane rupture during lytic cell death

Although report emerged as early as 2009 suggesting that NINJ1 mediates macrophage-induced programmed cell death during the early stages of ocular development by enhancing cell-cell and cell-matrix adhesions of macrophages (19), this does not appear to be the core mechanism underlying NINJ1-mediated cell death. In a groundbreaking study published in 2021, Kayagaki et al. demonstrated that NINJ1 plays a crucial role in PMR during lytic cell death (6). PMR is the ultimate catastrophic event of lytic cell death (20, 21). PMR releases intracellular molecules and cellular contents to trigger local inflammation (22). The mechanism of PMR has long been unknown and is generally regarded as a passive event following cell death. Kayagaki et al. discovered that PMR is mediated by NINJ1 (6), adding a novel regulatory step that is conserved across different types of lytic cell death, such as pyroptosis, necroptosis, and apoptosis, as well as ferroptosis, parthanatos, cuproptosis and H_2_O_2_-induced necrosis (23). These findings significantly expand our understanding of inflammatory cell death, indicating that the ultimate stages of membrane rupture are not simply passive processes but are actively governed. This revelation paves the way for the development of novel targeted therapeutics in clinical settings.

The control of PMR by NINJ1 was initially identified in pyroptosis (6). Pyroptosis is typical lytic cell death, a potent inflammatory mode of lytic cell death triggered by a variety of infectious and sterile insults, known as pathogen-associated molecular patterns (PAMPs) and damage-associated molecular patterns (DAMPs) (21, 22, 24). PAMPs and DAMPs are recognized by pattern recognition receptors, which assemble into caspase-1-activating protein complexes known as canonical inflammasomes (25). In humans, caspase-4/5, and in mice, caspase-11, can directly interact with intracellular lipopolysaccharide (LPS) to form “noncanonical inflammasomes,” leading to their activation. Once activated, caspase-1, -4, -5, and -11 cleave a pore-forming protein called gasdermin D (GSDMD) (26). The pore-forming fragment of GSDMD drives the process, ultimately releasing two key proteins: the pro-inflammatory cytokine interleukin-1β (IL-1β) and lactate dehydrogenase (LDH), which serves as a standard marker of PMR and lytic cell death (27–30). A seminal study proposed a two-step mechanism for pyroptosis: the initial formation of a small plasma membrane pore that facilitates the release of IL-1β and allows non-selective ionic fluxes, and the subsequent PMR, which is characterized by oncotic cell swelling (31). Although the estimated size of gasdermin pores, approximately 18 nanometers in inner diameter, is sufficient to permit the release of IL-1β (17 kDa, with a diameter of about 4.5 nm), the mechanism behind the subsequent PMR has traditionally been regarded as a passive osmotic lysis event, resulting in the release of LDH (140 kDa) and large DAMPs (32). The first step is generally believed to be regulated by GSDMD, while the second step has recently been confirmed to be controlled by NINJ1 (6). Kayagaki et al. found that upon pyroptosis signal stimulation, the release of LDH and large dextran dyes (150 or 70 kDa) was reduced when NINJ1 was deficient, without affecting the GSDMD-dependent release of a smaller dye (DD-3) or the intake of the 1.2 kDa cell-impermeable dye YOYO-1. Additionally, the deficiency of NINJ1 was dispensable for the production of the N-terminal pore-forming fragment of GSDMD caused by pyroptosis signals and the release of IL-1β, IL-18, IL-6, and TNF-α through the GSDMD pore. Morphologically, distinct morphological transformations are observable during the process of pyroptosis. Affected cells cease their movement, undergo swelling, and exhibit bubble-like protrusions that suddenly disintegrate, leading to the formation of a shrunken cell corpse (21, 31, 33). The absence of NINJ1 protein impedes the rupture of these vesicles while leaving the upstream events unaffected. Consequently, processes such as PMR and associated phenomena, including LDH release and vesicle lysis, are genetically distinct from GSDMD-mediated cell death and IL-1β secretion. This regulatory mechanism has been confirmed to be under the control of NINJ1.

**Figure 1:**
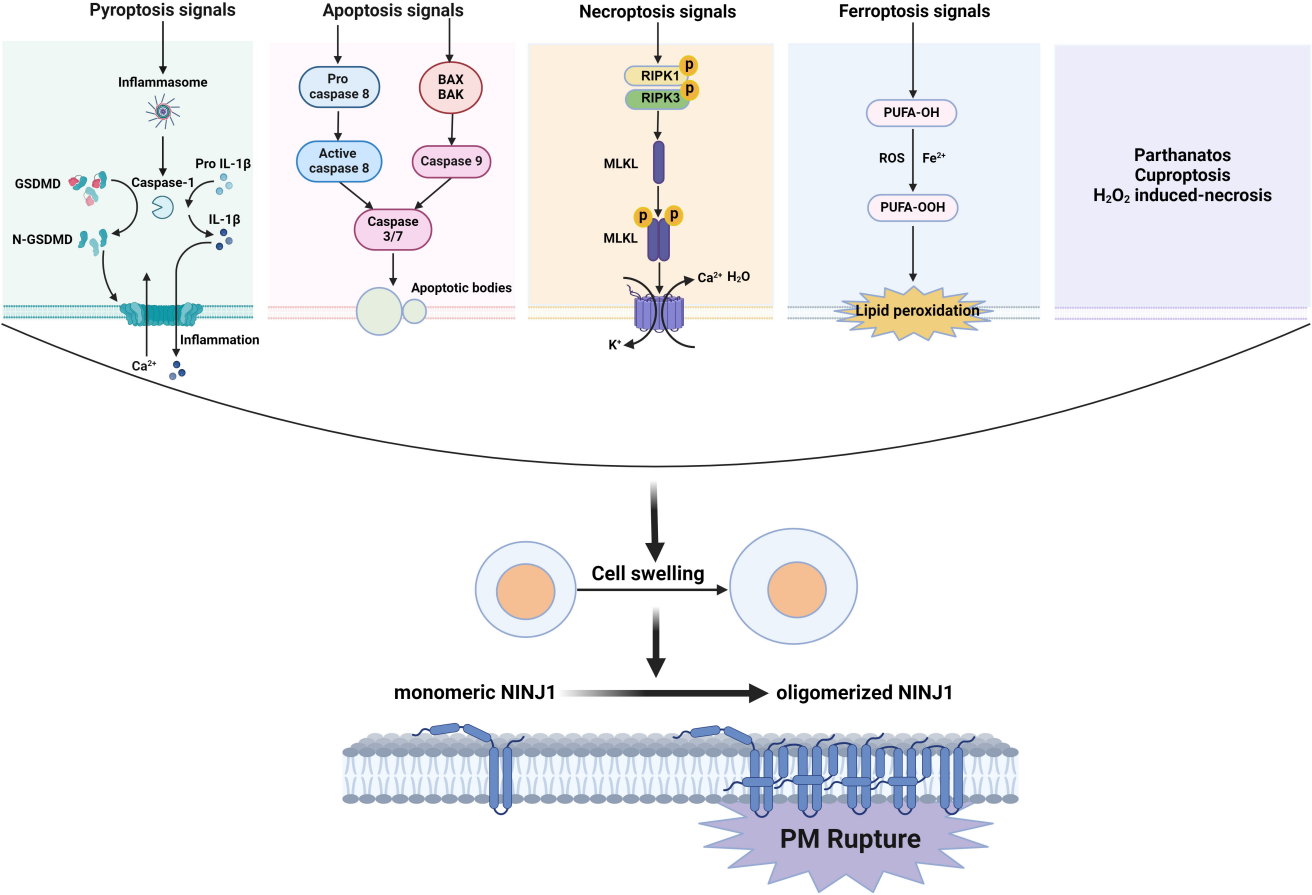
NINJ1 oligomerizes to induce PMR during lytic cell death. The signals of lytic cell death including pyroptosis, necroptosis, and apoptosis, as well as ferroptosis, parthanatos, cuproptosis, and H_2_O_2_-induced necrosis, drive a cell swelling, then induced monomer NINJ1 oligomerizes on the cell membrane resulting in PMR.

Not only is pyroptosis confirmed to have a global role in plasma membrane rupture (PMR) induced by other types of lytic cell death such as necrosis, post-apoptosis, necroptosis (6), PANoptosis (34), ferroptosis, parthanatos, cuproptosis and H_2_O_2_-induced necrosis (23), but NINJ1 has also been so established. In response to necrotic stimuli (such as bacterial pore-forming toxins) or apoptotic stimuli (like chemotherapeutic agents), NINJ1 deficiency attenuates the release of LDH, HMGB1, and other proteins, although it is ineffective in freeze/thaw-induced necrosis. Meanwhile, the absence of NINJ1 does not prevent the typical bubble formation of apoptotic cells but rather leads to the dead cells forming a durable balloon-like form without bursting (6). The role of NINJ1 in PMR in necroptosis currently seems ambiguous. Kayagaki et al. demonstrated that the deficiency of NINJ1 only partially mitigated the release of LDH and DNA degradation product DD-150 from BMDMs undergoing MLKL-dependent necroptosis (35, 36) following treatment with TNF plus the pan-caspase inhibitor zVAD (6). Furthermore, Dondelinger et al. found that NINJ1 did not contribute to the PMR during necroptosis in mouse embryonic fibroblasts (MEFs) or RAW264.7 cells (23). These findings collectively suggest that NINJ1 is unlikely to be the sole mediator of PMR. It is plausible that the oligomerization of MLKL itself directly disrupts the plasma membrane to trigger PMR, thereby obviating the requirement for NINJ1, or the PMR in necroptosis is controlled by other molecules. In PANoptosis induced by LPS plus heat shock, the mRNA and protein expressions of NINJ1 were significantly elevated. The deficiency of NINJ1 led to reduced cell death and a diminished release of inflammatory DAMPs, such as HMGB1 and LDH. Notably, the effect of NINJ1 on this process operates independently of the conventional lytic cell death executors, GSDMD, GSDME, or MLKL (23, 34). In ferroptosis, lipid peroxidation increases membrane tension and leads to cell rupture (37). NINJ1 oligomerization was observed during ferroptosis (38). After ferroptosis induction by the GPX4 inhibitors ML162 and RSL3, NINJ1 deficiency inhibited both Sytox Green positivity and LDH release. Ramos et al. also confirmed that NINJ1 controls ferroptosis-induced membrane permeabilization and cell lysis, as well as the release of DAMPs from ferroptotic cells (38). These findings indicated that NINJ1 controls the PMR and the release of both small and larger molecules in ferroptosis (23). Additionally, NINJ1-dependent PMR was also observed during parthanatos, cuproptosis, and H_2_O_2_-induced necrosis. Together, NINJ1 promotes PMR during almost all forms of lytic cell death, thereby broadening its significance in cell death. The above has been visualized as **Figure 1**.

The precise mechanism through which NINJ1 mediates the PMR remains elusive; however, it is likely intricately linked to its distinctive structural attributes. Although NINJ1 is widely recognized as an adhesion molecule, its adhesive segment appears dispensable for PMR. NINJ1 is an approximately 16 kDa monomer, but in response to nigericin or cytoplasmic LPS, it undergoes a transition from approximately 40 to 900 kDa, suggesting that NINJ1 exists as a dimer or trimer in unstimulated BMDMs and further oligomerizes upon exposure to death stimuli (6). Degen et al. have subsequently elucidated the detailed structural basis of NINJ1 and the mechanism by which it ruptures membranes (7). They noted that following inflammasome activation, NINJ1 dimers, and trimers began to form within 10 minutes, which was followed by a significant aggregation of NINJ1, resulting in the creation of larger polymers at subsequent time intervals and aggregation at the plasma membrane. Utilizing stochastic optical reconstruction microscopy, they explored the nanoscale arrangement of NINJ1 aggregates within live cells. These cells exhibited a uniform distribution of minute NINJ1 dots and a few compact, generally circular clusters (7). In contrast, cells undergoing pyroptosis exhibited more prominent NINJ1 clusters. In pyroptotic cells, NINJ1 undergoes polymerization, giving rise to large clusters with various shapes, including branched, filamentous structures, as well as elongated filaments reaching into the micrometer scale. These filaments can be distinctly modeled as consisting of four α-helices designated α1–α4 (7). In viable cells, NINJ1 exists as a monomer embedded in the cellular membrane, with helices α1 and α2 located on the extracellular surface, while the α3 and α4 pair is integral to the membrane. During the process of cell death, the amphipathic helices α1 and α2 insert into the membrane, adopting a kinked conformation that facilitates the bridging of adjacent protomers, thereby creating larger polymer structures. These higher-order polymeric formations contribute to membrane disruption by effectively sealing the membrane edges (7). Subsequently, David et al. employed a combination of structural biology and cellular imaging to present a different model regarding NINJ1-mediated PMR (17). In contrast to the conclusions of Degen et al., David et al. observed that α3 and α4 are both kinked rather than straight (17). They further discovered that recombinant NINJ1 alone is sufficient to dissolve liposomes into heterogeneous, ring-like NINJ1-containing assemblies. Live-cell imaging of NINJ1 KO human THP-1 cells reconstituted with NINJ1-GFP revealed the release of NINJ1 rings into the culture media during NLRP3 inflammasome activation, and western blot analysis showed the abundance of NINJ1 oligomers in the culture supernatant but not in the cell pellets. Thus, these data suggest the rather surprising possibility that NINJ1 ruptures membranes by cutting them into small pieces, leading to lytic cell death and a more complete release of DAMPs. Glycine has historically been known for its ability to safeguard cells against PMR caused by a variety of harmful stimuli, including lytic cell death (39, 40). However, the molecular target and the underlying mechanism by which glycine confers this cellular protection have remained elusive. Borges et al. demonstrated that the knockout or silencing of NINJ1 in both mouse and human primary macrophages replicates the cytoprotective effects of glycine, both functionally and morphologically, when cells are stimulated to undergo different forms of lytic cell death (41). Moreover, they showed that treatment with glycine inhibits the clustering of NINJ1 within the plasma membrane, thereby maintaining membrane integrity. The results indicated that NINJ1-dependent PMR is a target of glycine. Similarly, muscimol, a small molecule that prevents PMR during pyroptosis, whose mechanism was previously unidentified, has been shown to block oligomerization of NINJ1, which is essential for PMR, and thus reduces lethality during LPS-induced septic shock (42). Together, these findings demonstrate that NINJ1-mediated PMR can be pharmacologically manipulated and lay the groundwork for the identification of therapeutic strategies for pathologic conditions.

As PMR represents the terminal event in lytic cell death, it is believed that NINJ1 plays a crucial role in the final stages of cell death. The deficiency of NINJ1 does not affect metabolic cell death induced by executors such as GSDMD, GSDME, MIKL, and Caspase 3, suggesting that NINJ1 functions downstream of these molecules (23). Additionally, NINJ1 has also been demonstrated to be located downstream of lipid peroxidation in the process of ferroptosis (23). These findings imply that NINJ1 regulates plasma membrane permeabilization downstream following metabolic cell death. The signaling cascade triggering NINJ1 was once shrouded in mystery. Pourmal et al. identified NINJ1 autoinhibition as the mechanism that prevents unprovoked PMR through structure–function findings (43). They illustrated that dimeric NINJ1 sequesters the hydrophilic face and that the dissociation of face-to-face dimers is essential for NINJ1 activation. They proposed a possibility of a two-step activation model for NINJ1: In the first step, the inactive-state N-out NINJ1 inserts its transmembrane domain 1 (TM1) into the membrane to form intermediate-state N-in face-to-face dimers; in the second step, the N-in face-to-face dimers dissociate, and TM1 kinks to stabilize the active-state NINJ1 multimers (43). Kayagaki et al. hypothesized that an increase in cell size or cell swelling could be a pivotal factor, but there was a lack of direct evidence at that time (6). This conjecture has since been validated by Dondelinger et al. They exposed wild-type and NINJ1-deficient MEFs to a hypotonic shock by reducing the medium’s osmolarity from 360 mOsm to 76 mOsm. Notably, this hypotonic environment was sufficient to induce NINJ1 oligomerization and NINJ1-dependent PMR without affecting metabolic cell death (23). These results indicate that NINJ1 oligomerization does not necessarily lead to NINJ1-dependent PMR; rather, it is associated with cell swelling. Furthermore, Zhou et al. revealed that Ca^2+^ may be an activator of NINJ1. They observed that the presence of Ca^2+^ chelators (BAPTA-AM) almost completely inhibited the oligomerization of NINJ1 and thus stabilized platelet morphology (44).

The role of NINJ1-mediated PMR in disease remains virtually unknown, with only a few studies initially exploring it. After discovering NINJ1-mediated PMR, Kayagaki et al. subsequently constructed an anti-NINJ1 monoclonal clonal antibody that specifically targets mouse NINJ1 and blocks the oligomerization of NINJ1, thereby preventing PMR. Through the use of these antibodies to inhibit NINJ1, hepatocellular PMR induced by TNF plus D-galactosamine, concanavalin A, Jo2 anti-Fas agonist antibody, or ischemia-reperfusion injury was alleviated (45). Moreover, NINJ1 plays an important role in inflammation and lethality downstream of PMR. Inhibiting NINJ1-dependent PMR protects against LPS-induced sepsis and inflammasome-induced blood coagulation and inflammation (42, 46), as well as platelet activation and PANoptosis in septic disseminated intravascular coagulation (44). Similar effects of NINJ1 have been confirmed in infection conditions and heat stress (34). These results suggest a crucial role for NINJ1-dependent PMR in cell death and disease.

Despite the significant advancements made in understanding NINJ1-mediated PMR in lytic cell death, the relatively brief history of research in this area has left ample room for further exploration. For instance, the precise mechanism by which NINJ1 is activated in the context of cell death remains to be elucidated. Additionally, the ambiguous role of NINJ1 in necroptosis calls for a closer examination of both the similarities and differences in NINJ1’s regulation of PMR across various cell death modalities. Crucially, the implications of NINJ1 in disease-related PMR regulation are yet to be fully grasped, and the viability of therapeutic strategies targeting NINJ1 requires validation.

## 4 NINJ1 regulated immune response in inflammatory diseases

NINJ1 is initially identified as an adhesion molecule in the CNS that is crucial for cell-to-cell interactions. Subsequently, it has been observed to be highly expressed in various types of immune cells such as macrophages/monocytes and neutrophils, suggesting the regulatory potential of NINJ1 for immune and inflammatory diseases (19, 47–49). Owing to the adhesion activity of NINJ1, NINJ1 enhances the adhesion between immune cells and vascular endothelial cells, thereby playing a key role in nervous system inflammation (8, 9, 50, 51) and peripheral inflammatory diseases like pulmonary fibrosis and atherosclerosis (52, 53). Notably, NINJ1 has been found by Jennewein et al.” and “Shin et al. to directly target toll-like receptor 4 (TLR4) to regulate immunity in sepsis (54, 55). Additionally, NINJ1 has recently been discovered to mediate PMR in lytic cell death and thus may regulate immunity and related diseases by regulating the release of DAMPs and inflammatory factors (34, 56, 57). In summary, the regulatory mechanisms of NINJ1 on immune and inflammatory diseases may be multifaceted.

Since NINJ1 was initially discovered in the central nervous system, its role in immune and inflammatory diseases was initially revealed in neuroinflammation. Ahn et al. and Ifergan et al. noted that NINJ1 is predominantly expressed in the meninges, the choroid plexus, and the parenchymal perivascular region of normal rat brains, while being strongly expressed in myeloid cells and partially expressed in endothelial cells (ECs), but not lymphocytes or astrocytes (8, 9). Functionally, NINJ1 mediates adhesion between monocyte lineage cells and ECs, suggesting that NINJ1 might enhance myeloid cell trafficking into the brain across the blood-brain barrier (BBB). NINJ1 neutralization specifically abolished the adhesion and migration of human monocytes across BBB-ECs without affecting lymphocyte recruitment. NINJ1 blockade or deficiency attenuated the susceptibility to EAE by reducing leukocyte recruitment, macrophage infiltration, dendritic cells, and antigen-presenting cells into the CNS (9, 50). Mechanistically, the ability of NINJ1 to enhance the basal motility and transendothelial migration of immune cells is achieved by inducing protrusive membrane dynamics (51). Additionally, the ability of NINJ1 to regulate inflammation has also been demonstrated in inflammatory diseases outside the central nervous system. During the development of intestinal inflammation, the increased NINJ1 induces the activation of macrophages or aberrant M1/M2 macrophage polarization, and dysbiosis contributes to the pathogenesis of inflammatory bowel disease (58, 59). NINJ1 may contribute to the activation of macrophages by enhancing their interaction with epithelial cells, thereby facilitating neutrophil infiltration, inducing local inflammation, and promoting apoptosis in pulmonary fibrosis and liver ischemia-reperfusion injury (52, 60). The regulatory mechanism of NINJ1 in systemic inflammation appears to be somewhat distinct. In sepsis, Jennewein et al. discovered that inhibiting NINJ1 can alleviate systemic and pulmonary inflammation and organ damage, and enhance the survival rate at 24 hours. Its mechanism not only inhibits leukocyte migration but also potentially reduces the expression of TLR4-dependent inflammatory mediators. Subsequently, Shin et al. further confirmed that NINJ1 regulates LPS-induced inflammatory responses by directly binding to LPS, thus suggesting a new mechanism and potential for NINJ1 as a hypothetical target for treating inflammatory diseases.

PMR and cell death play a critical role in inflammatory responses, which control the release and diffusion of various DAMPs, including pro-inflammatory cytoplasmic molecules (6). NINJ1 has been reported to regulate the immune response by mediating the PMR in dying cells. During Yersinia infection, the loss of NINJ1 leads to susceptibility, disrupting the macrophages’ ability to undergo PMR downstream of gasdermin cleavage and affecting host survival and bacterial control (56). In severe acute pancreatitis (SAP), Ca^2+^ overload induces mitochondrial stress, thereby upregulating the P53/NINJ1 pathway and inducing PMR of acinar cells to exacerbate the severity of SAP (12). During severe infections such as sepsis and COVID-19, NINJ1 regulates the activation and PMR of platelets and the release of procoagulant microvesicles to promote the formation of thrombosis and disseminated intravascular coagulation (44, 46). After Kayagaki et al. first demonstrated that NINJ1 mediates PMR in lytic cells, they generated NINJ1-blocking antibodies that can block the oligomerization of NINJ1 to prevent PMR (6). They further confirmed that the NINJ1 antibody can ameliorate TNF + D-galactosamine, legumin A, Jo2 anti-FAS agonist antibody, or ischemia-reperfusion injury-induced PMR in hepatocytes, suggesting that NINJ1 mediates PMR and inflammation in diseases driven by abnormal liver cell death (45).

## 5 Role of NINJ1 in neurological diseases

In 1996, NINJ1 was initially identified as a gene regulated in Schwann cells following nerve injury and was named NINJ1 (for nerve injury-induced protein) by Araki et al. (1). Subsequently, more roles of NINJ1 in the nervous system were gradually unveiled, mainly encompassing mediating nerve and cerebrovascular repair and regeneration as well as neuroinflammation. NINJ1 was initially identified by Araki et al. as a novel adhesion molecule induced by nerve injury that can facilitate axonal growth (1). To identify the genes promoting nerve regeneration, Araki et al. employed differential screening strategies to screen the genes regulated in Schwann cells from the distal stump of sciatic nerves. They observed that NINJ1 was upregulated after axotomy in neurons and Schwann cells surrounding the distal nerve segment. NINJ1 is located on the cell surface and is capable of mediating homophilic adhesion and promoting the neurite extension of dorsal root ganglion neurons *in vitro*. In cavernous nerve injury, high-dose NINJ1-Ab induced a profound restoration of erectile function while low-dose NINJ1-Ab elicited partial improvement, indicating the dual neurotrophic and angiogenic effects of NINJ1 blockade in treating cavernous nerve injury (61). Similarly, following sciatic nerve crush injury, a substantial increase in NINJ1 promotes the expression of the myelin binding protein and also increases the number of myelinated axons. In the postischemic brain, the NINJ1 N-terminal adhesion motif stimulates the proliferation, migration, and tube formation of HUVECs by activating the Angiopoietin-1 (Ang1)-Tie2 and AKT signaling pathways. In a rat MCAO model, N-NAM augmented angiogenesis in the penumbra of the ipsilateral hemisphere of the brain and significantly enhanced total vessel lengths, vessel densities, and pro-angiogenic marker expression. This confers the pro-angiogenic effects of NINJ1 and implies that these effects might contribute to its neuroprotective effects in the postischemic brain (4). Additionally, in the previous part, we also elaborated on the regulatory effect and mechanism of NINJ1 on neuroinflammation. In conclusion, NINJ1 can fulfill its role in the nervous system by mediating the repair and regeneration of nerves and blood vessels as well as neuroinflammation, suggesting its potential as a potent therapeutic target for neurological diseases.

## 6 Role of NINJ1 in cancer

NINJ1 expression is induced in response to various stresses within the tumor microenvironment (62, 63) and it plays an important role in macrophage-mediated inflammation and vascular remodeling (19, 64), both of which are closely associated with cancer development and progression (65). Additionally, NINJ1 is overexpressed in various cancers, such as hepatocellular carcinoma (49), acute lymphoblastic B-cell leukemia (48), urothelial bladder cancer (66), and circulating prostate cancer cells (67). Furthermore, NINJ1 has been identified as a novel prognostic and severity predictor in human hepatocellular carcinoma (49, 68), serous ovarian cancer (69), and retroperitoneal liposarcoma (70). However, the mechanism by which NINJ1 functions in tumorigenesis, metastasis, and cancer progression remains not fully understood. p53, a crucial tumor suppressor protein, contributes to the regulation of NINJ1 in cancer (63, 71). Mechanically, during the process of tumorigenesis, DNA damage transcriptionally regulates NINJ1 in a p53-dependent manner. The deficiency of NINJ1 increases p53 expression potentially by enhancing p53 mRNA translation to suppress cell proliferation but enhances apoptosis and premature senescence (63). Interestingly, the loss of NINJ1 led to an increase in mutant p53 expression and subsequently enhanced cell growth and migration in cells carrying a mutant p53. Conversely, loss of NINJ1 inhibited cell growth and migration in cells with a WT p53 (71). Taken together, NINJ1 may exhibit two opposing functions in tumorigenesis depending on the p53 genetic status. Moreover, due to its adhesion properties, NINJ1 is effective in facilitating tumor metastasis in multiple tumor types. In irradiated xenograft tumors, NINJ1 contributes to the recruitment of monocytes in the tumor and the adhesion of endothelial cells and monocytes (72), and this process is still transcriptionally regulated by p53. Similarly, in the colitis-mediated colon cancer mouse model, NINJ1 decreases macrophage migration into the tumor sites, thereby reducing macrophage infiltration and suppressing angiogenesis in the tumor mass, resulting in the development of fewer and smaller tumors (73). In lung cancer, inhibiting NINJ1 significantly increased the expression and secretion of IL-6, enhanced cell migration, and ultimately induced a significant increase in the incidence of lung metastasis, as well as the sizes and number of tumor nodules, without affecting tumor growth (74). In summary, NINJ1 may promote monocyte/macrophage migration to tumor sites in a p53-dependent manner, thereby exacerbating tumorigenesis and metastasis.

## 7 Role of NINJ1 in vascular diseases

Multiple studies have unveiled the crucial role of NINJ1 in inflammatory response and angiogenesis in vascular diseases. The soluble form of NINJ1 is an antiatherogenic protein, and its plasma levels are elevated in patients with coronary artery disease (CAD) and are associated with the occurrence of CAD and the severity of coronary stenosis (75). NINJ1-deficient macrophages promoted the expression of proinflammatory genes by activating the mitogen-activated protein kinase and inhibiting the phosphoinositide 3-kinase/Akt signaling pathway. Whole-body and bone marrow-specific NINJ1 deficiencies significantly increased the recruitment of monocytes and the accumulation of macrophages in atherosclerotic lesions through enhanced macrophage-mediated inflammation. As a substrate of matrix metalloproteinase 9, macrophage NINJ1 is directly cleaved by matrix metalloproteinase 9 to generate a soluble form, which reduces the expression of proinflammatory genes in human and mouse classically activated macrophages, thereby attenuating monocyte transendothelial migration and alleviating atherosclerosis (76). Pericyte-derived NINJ1 plays an important role in angiogenesis. Matsuki et al. identified NINJ1 as a candidate factor for angiogenesis regulation through a microarray screen. They observed that NINJ1 is expressed in capillary cells, including endothelial cells (ECs), and is expressed at a higher level in capillary pericytes (cPCs), which can be further induced by hypoxia. The downregulation of NINJ1 enhanced the production of angiogenic growth factors such as VEGF and angiopoietin 1, and the angiogenic effects mediated by cPCs (62).

Similarly, in response to ischemia, NINJ1 enhanced the formation of functional matured vessels through the association between pericytes and ECs, resulting in blood flow recovery from ischemia (77). After a traumatic vascular injury, the deletion of NINJ1 in pericytes induces the formation of immature vasa vasorum in the injured vasculature and exacerbates adventitial inflammation and intimal hyperplasia (78). In diabetes-induced vascular degeneration and endothelial dysfunction, the administration of a NINJ1-neutralizing antibody reversed the downregulation of Ang1 expression and the upregulation of angiopoietin-2 expression, successfully restoring erectile function through enhanced penile angiogenesis and neural regeneration (79, 80). Additionally, the functional blocking of NINJ1 exerts protective effects on diabetic endothelial cells by inhibiting the caspase 3-dependent apoptosis pathway (81). In conclusion, inhibiting NINJ1 may be a novel therapeutic strategy for treating vascular diseases by regulating the endothelial inflammatory response, angiogenesis, and apoptosis.

## 8 Conclusions and prospects

NINJ1 was originally identified as an adhesion molecule induced by nerve injury to promote axon growth (1). Subsequent studies have shown that NINJ1 is the least expressed in the CNS but abundant in the skin and ileum and is moderately expressed in the sciatic nerve, spleen, lung, stomach, colon, liver, pancreas, kidney, testis, and other tissues in mice (11). Researchers have therefore attempted to investigate NINJ1’s role outside the CNS. Owing to its adhesion properties, NINJ1 also plays an important role in inflammatory diseases (16), cancer (70), and vascular injuries (75). However, these discoveries are not sufficient to truly highlight NINJ1’s significance. In 2021, Kayagaki et al. screened for PMR in which NINJ1 could mediate lytic cell death (6) and transformed the previously uncontrollable PMR process into a procedural stage regulated by NINJ1. Subsequent studies further unveiled the structural basis and mechanism of NINJ1’s regulation of PMR (7). NINJ1’s four α-helical structures mediate the oligomerization of NINJ1 following stimulation by lytic cell death signals, a modified form that causes NINJ1 to induce PMR. Additionally, the NINJ1 antibody has been proven to reduce PMR by inhibiting the oligomerization of NINJ1 (45), ultimately alleviating a variety of liver injury diseases, indicating the potential of inhibiting NINJ1-mediated PMR as a therapeutic target for diseases. Nevertheless, there are still numerous unanswered questions waiting to be revealed in future research. NINJ1 is believed to play a role in the final stage of cell death, but the signal of NINJ1 activation is still unclear, and cell swelling and Ca^2+^ are considered to be possible factors, but more direct and sufficient evidence is still needed to prove it. NINJ1 is believed to be involved in the final stage of cell death, yet the signal for its activation remains unclear. Cell swelling and Ca^2+^ are considered possible factors, but more direct and sufficient evidence is still required to substantiate this. Although Kayagaki et al. have established a NINJ1-neutralizing antibody and demonstrated its positive role in liver injury diseases (6), the role of NINJ1-mediated PMR in other diseases still requires further exploration, and the development of NINJ1-targeted therapeutic approaches holds great clinical translational value. Notably, in addition to the more commonly studied areas such as neurological diseases, tumors, inflammatory diseases, and vascular damage, the role of NINJ1-mediated PMR in infection is difficult to determine. NINJ1-mediated PMR leads to the release of intracellular substances, which may either amplify the inflammatory response or actively promote an adaptive protective response. This could be a point that NINJ1 cannot overlook in its clinical transformation. In addition to cell membranes (6), NINJ1 localization has also been detected on intracellular organelle membranes such as mitochondria, lysosomes, and the endoplasmic reticulum (7, 12). Therefore, whether NINJ1 has a similar regulatory effect on the breakdown of organelle membranes is worthy of determination in future studies, and it may play an important role in mitochondrial, lysosome damage, and other cellular processes. In conclusion, in addition to its known adhesion properties, the PMR in NINJ1-mediated lytic cell death unfolds a new and broader realm for NINJ1 research, which is bound to thrive, making NINJ1 a potential therapeutic target for an even greater number of diseases in the future.
